# Promoting the practice of exclusive breastfeeding: a philosophic scoping review

**DOI:** 10.1186/s12884-022-04689-w

**Published:** 2022-05-01

**Authors:** Tumilara Busayo Amoo, Tosin Popoola, Ruth Lucas

**Affiliations:** 1 School of Nursing, University of Connecticut, Storrs, CT USA; 2 School of Nursing, Victoria University of Wellington, Wellington, New Zealand

**Keywords:** Exclusive breastfeeding, Practice, Philosophy, Scoping review, Theory

## Abstract

**Background:**

The World Health Organization recommends exclusive breastfeeding for the first 6 months of an infant’s life and continued breastfeeding for 2 years. The global rate of exclusive breastfeeding is low at 33%. Thus, it is important to identify philosophical and theory-based strategies that can promote exclusive breastfeeding. The aim of the study was to identify philosophical schools of thought and theories used in research on promoting the practice of exclusive breastfeeding.

**Methods:**

A scoping review using Arksey and O'Malley's framework explored the phenomenon of exclusive breastfeeding practice promotion. Searches were conducted using CINAHL Plus full-text, PubMed, APA PsycInfo, and Academic Search Premier. Search terms included theory, philosophy, framework, model, exclusive breastfeeding, promotion, support, English, and publication between 2001—2022.

**Results:**

The online search yielded 1,682 articles, however, only 44 met the inclusion criteria for the scoping review. The articles promoting exclusive breastfeeding used pragmatism (*n* = 1) or phenomenology (*n* = 2) philosophies and theories of self-efficacy (*n* = 10), theory of planned behaviour (*n* = 13), social cognitive theories (*n* = 18) and represented 16 countries. Theories of self-efficacy and planned behaviour were the most used theories.

**Conclusions:**

This review suggests that theories and models are increasingly being used to promote exclusive breastfeeding. Orienting exclusive breastfeeding programmes within theoretical frameworks is a step in the right direction because theories can sensitize researchers and practitioners to contextually relevant factors and processes appropriate for effective exclusive breastfeeding strategies. Future research should examine the efficacy and effectiveness of theory-informed exclusive breastfeeding programmes over time. Such information is important for designing cost-effective EBF programmes.

**Supplementary Information:**

The online version contains supplementary material available at 10.1186/s12884-022-04689-w.

## Background

Exclusively breastfeeding infants for 6 months is the global public health gold standard [1, 2] because of its benefits for infants, women, and the society [3, 4]. For example, exclusively breastfed infants have higher cognitive developmental scores, have reduced risk of gastrointestinal and respiratory diseases, and are less likely to develop lifelong obesity and diabetes [5–7]. Similarly, exclusive breastfeeding (EBF) promotes healthy weight, prolongs lactational amenorrhoea and reduces the risk of breast cancer among women [8, 9]. The benefits of EBF are also enormous for the society. As an illustration, EBF is not only cost-effective, but it also decreases parental absenteeism from work and reduces the burden of formula cans on the environment [10]. Research from United Kingdom also suggested that if all infants were breastfed, a total lifetime cost savings to the National Health Service would be £46.7million and a total lifetime quality-adjusted life year (QALY) gain of 10,594 [11]. Additional research from Canada reported cost savings of $13,812 per additional QALY gained [12].

Despite these benefits, there has been little improvement in the global practice of EBF in two decades. For example, only 1 out of 3 children received EBF for 6 months [1]. Exclusive breastfeeding rates at 6 months differ across the globe, varying from 1% in the UK [13] to 69% in Peru [14]. The low rates of EBF (< 50%) at 6 months in many countries across the globe have been studied. Research suggests that lack of support from husbands, fear of infants becoming addicted to breast milk [15], non-approval from family members and maternal or infant lack of strength due to inadequate nutrition [16], lack of capacity to store human milk [17], lack of institutional and family support [18], and unfavourable work conditions [19] are barriers to EBF. Because of the benefits of EBF for infants, women and societies, many interventions have been implemented for the purpose of increasing the adoption of EBF practice [20, 21]. Many of these interventions are a combination of baby friendly initiatives and provider led initiatives. However, there is limited information about the philosophical worldviews underpinning these interventions. EBF interventions like any intervention can be better understood and evaluated if the underlying philosophical thoughts of such programmes are understood. In view of the above, this study aimed to identify and evaluate the philosophies and theories used in research to promote exclusive breastfeeding through a scoping review [22]. Such information is important to inform clinical practice and improve knowledge.

Scoping reviews are ideal to determine the breadth of a body of literature on a topic of interest, identify and analyse knowledge gaps, clarify key concepts in literature, map features of primary research, and act as a precursor to focused systematic reviews [23, 24]. Previous scoping reviews have identified breastfeeding social support models using Arksey and O’Malley’s framework [25, 26]. However, these studies focused on any breastfeeding -breast milk in addition to food and other fluids [27, 28] and did not provide the philosophical schools of thought or theories underlying those models. No study has investigated theories and/or philosophies used to support interventions to promote EBF. Therefore, this scoping review will fill the knowledge gap. The primary aim of this study was to identify and evaluate the philosophies and theories used in research to promote exclusive breastfeeding practice, to inform clinical practice and improve knowledge.

## Methods

A scoping review following Arksey and O’Malley’s framework explored the phenomenon of EBF practice promotion. This framework has five stages: Identifying the research objectives, identifying relevant studies, study selection, charting the data, and collating, summarizing. and reporting the results [23]. A systematic literature search for relevant articles was conducted across four databases, PubMed, CINAHL Plus with full-text, APA PsycInfo, and Academic Search Premier. The search was conducted using text words in various combinations relating to promotion of EBF. The key search terms were breast feeding, breast-feeding, breastfeeding exclusivity, enhance, exclusive breastfeeding, increase, improve, promoting, promotion, philosophy, support, theory, model, and framework, see Table 1 for search strategy.

**Table 1:** Search strategy

Database	Search strategy
CINAHL Plus with full text	• (Theory OR Philosophy OR Framework OR model) AND (Exclusive breastfeeding OR exclusive breast feeding OR exclusive breast-feeding OR breastfeeding exclusivity) AND (promotion OR promote OR promoting OR enhance OR improve OR increase OR support) *(n* = *546)*
PubMed	• ((((Theory[Title/Abstract]) OR (Philosophy[Title/Abstract])) OR (Framework[Title/Abstract])) OR (Model[Title/Abstract]) AND ("exclusive breastfeeding"[Title/Abstract])) OR ("breastfeeding exclusivity"[Title/Abstract]) AND ((support [Title/Abstract]) OR (promoting[Title/Abstract]) OR (promotion[Title/Abstract])) *(n* = *282)* • ((((Theory[Title/Abstract]) OR (Philosophy[Title/Abstract])) OR (Framework[Title/Abstract])) OR (Model[Title/Abstract]) AND ("exclusive breastfeeding"[Title/Abstract])) OR ("breastfeeding exclusivity"[Title/Abstract]) AND ((promote[Title/Abstract]) OR (enhance[Title/Abstract]) OR (increase[Title/Abstract])) *(n* = *197)*
APA PsycInfo	• (Theory OR Philosophy OR Framework OR model) AND (Exclusive breastfeeding OR exclusive breast feeding OR exclusive breast-feeding OR breastfeeding exclusivity) AND (promotion OR promote OR promoting OR enhance OR improve OR increase OR support) *(n* = *165)*
Academic Search Premier	• (Theory OR Philosophy OR Framework OR model) AND (Exclusive breastfeeding OR exclusive breast feeding OR exclusive breast-feeding OR breastfeeding exclusivity) AND (promotion OR promote OR promoting OR enhance OR improve OR increase OR support) *(n* = *492)*

### Study selection criteria

Articles of interest were those that focused on the promotion of EBF, not just promotion of breastfeeding. Four inclusion criteria were used to select relevant articles including (1) focused on exclusive breastfeeding: The phenomenon of interest is exclusive breastfeeding-breast milk only and no other liquids or solids with the exception of drops or syrups consisting of vitamins, mineral supplements or medicines [29] (2) used philosophy/framework to address phenomenon: (3) published in English: Researchers prefer articles written in English for easy comprehension (4) published between 2001–2022: World Health Organization recommended exclusive breastfeeding for 6 months in 2001 (5) study methodology: quantitative and/or qualitative studies were included but review articles were excluded.

### Search outcomes

**Fig. 1:**
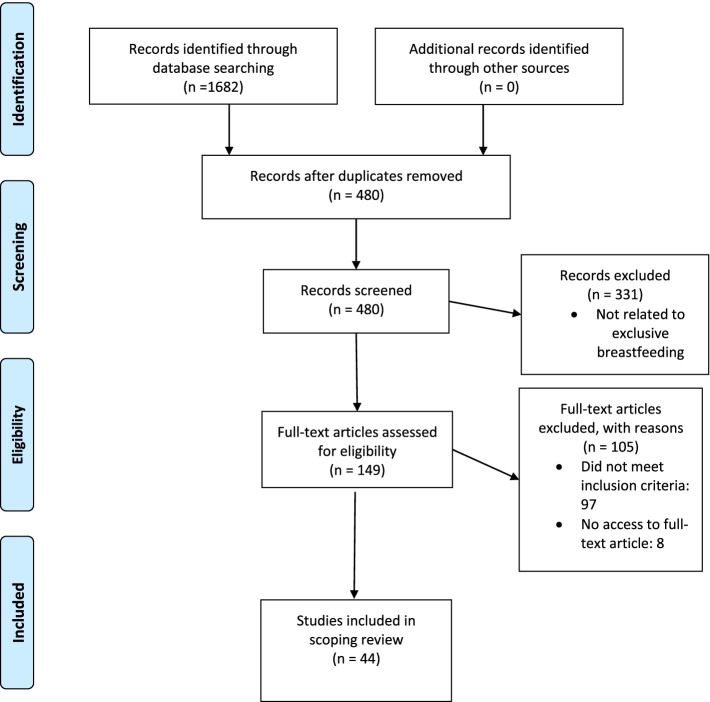
PRISMA flowchart of study selection process. Adapted from Moher et al (2009) [30]

The search identified 1,682 titles. After removal of duplicates, 480 articles underwent title/abstract screening, and 331 articles were excluded as they did not address exclusive breastfeeding promotion. Thus, 149 full-text articles were assessed for eligibility, and 52 articles were eligible for inclusion. The matching full-text articles were acquired for review. Eight articles could not be accessed and were not included in the review. Therefore, 44 articles were selected and included for analysis in the scoping review. Figure 1 (PRISMA flowchart) showed the process of article selection.

### Quality appraisal

Corresponding author assessed the quality of included studies using an adapted Critical Appraisal Skills Programme [CASP] checklist for randomised controlled trials (RCT) and qualitative studies. CASP RCT checklist consists of 4 sections containing 11 questions (see supplementary) [30]. Other quantitative studies were evaluated using Holland and Rees’ (2010) framework for critiquing quantitative research articles (see supplementary) [31]. CASP checklist for qualitative studies consists of 3 sections containing 10 questions that researchers need to ask when evaluating evidence from qualitative studies (see supplementary) [32]. Section A examines result validity, section B examines the entire results, and section C examines applicability of results. In this review, question 10 in the CASP qualitative checklist ‘How valuable is the research?’ was adapted as ‘Is the research valuable?’ for scoring to be completed. Similarly, question 11 in the CASP RCT checklist was adapted as ‘Would the experimental intervention provide value to the people in your care? Ten relevant questions from Holland and Rees’ (2010) framework for critiquing quantitative research articles were used to appraise other quantitative studies. Response to each question was given a score of 1. Studies with overall score of 7 or above were eligible for inclusion.

### Data extraction and analysis

TBA and TP conducted literature review, reviewed paper titles, and screened abstracts for eligibility to reduce subjectivity of analysis. Data from articles included in the scoping review were extracted manually using two templates developed by the first author. The first template contained general characteristics of the study, and the second template contained the philosophies and theories. Extracted information included study purpose, design, population characteristics, methods, philosophy or theoretical basis, and results. TBA and TP independently extracted data from the articles using the templates. In the case of disagreements, both authors reviewed study eligibility criteria and discussed reasons why the articles should or should not be included based on the criteria. At the end of discussion, consensus was reached on the article inclusion. Articles not related to exclusive breastfeeding promotion were excluded.

Extraction of the data continued until all the philosophies/frameworks and theories were identified. A table was then created to fit the extracted data. For this scoping review, studies were grouped based on similarities in philosophies and theoretical frameworks used to promote exclusive breastfeeding. A summary of the findings from the articles were presented and data were analysed using narrative synthesis. Narrative synthesis is the preferred method of data analysis in reviews of quantitative studies when it is not possible to conduct a statistical analysis [33]. The summaries in this scoping review illustrate the scope of evidence, rather than describing the quality of the studies. Ethical approval was not required for this scoping review.

### Overview of theories

Ten theories, two philosophies, four frameworks and eleven models were extracted. The goal of *theory of planned behaviour* (TPB) is to predict and explain behaviour. TPB and *Reasoned Action Approach* developed by Fishbein and Ajzen (2010) originated from the *theory of reasoned action*. *Reasoned action approach* posited that attitude towards behaviour, perceived norm, and perceived behavioural control, determine intention, which predicts behaviour [34]. Bandura’s *theory of self-efficacy* and Dennis’ *breastfeeding self-efficacy theory* also originated from Bandura’s (1986) *social cognitive theory* [35]. Bandura defined self-efficacy as the belief in a person’s ability to organize and accomplish actions required to manage prospective situations [36]. Self-efficacy influences thinking and decision-making, effort and persistence, and choice. Dennis defined breastfeeding self-efficacy as a mother’s perceived ability to breastfeed her infant [37]. One of the sources of self-efficacy is information received through verbal persuasion [38]. Hence, utilizing the breastfeeding self-efficacy theory, health professionals may be able to influence the practice of breastfeeding by modifying this information [37]. Health professionals can lead a change with a top-down approach using Kotter’s *theory of change* which was specifically designed to be applied in leadership. Kotter described eight steps in the process of change including creating a sense of urgency, forming guiding coalitions, vision development, communicating the vision, removing obstacles and employee empowerment, creating short-term wins, consolidating gains, and strengthening change by anchoring change in the culture [39]. Mann’s *adolescent decision-making competence theory* (ADM) suggests that competent decision-making has nine elements including choice, comprehension, creativity, compromise, consequentiality, correctness, credibility, consistency, and commitment [40].

Granovetter’s *strength of weak ties theory* posited that individuals’ personal experiences is embedded within the larger social structure beyond the control of some individuals [41]. In their theory, Milligan and Wiles described *landscapes of care* as the result of interaction between socio-structural processes and structures that shape experiences and practices of care [42]. In addition, Mercer affirmed the significance of social support in her *theory of maternal role attainment.* The theory suggested that maternal role attainment is influenced by maternal age, socioeconomic status, perception of birth experience, early mother-infant separation, social stress, social support, personality traits, self-concept, child-rearing attitudes, perception of infant, role strain, and health status [43]. Social norms are informal, acceptable standards of behaviour in a society which may affect an individual positively or negatively. However, social support encompasses resources (human and non-human) available to assist an individual in the society.

Theories of *self-efficacy, planned behaviour*, *maternal role attainment*, *adolescent decision-making* and *social cognitive theory* primarily emphasized individual factors that influence performance of a behaviour. On the other hand, theories of *strength of weak ties* and *landscapes of care* and *change theory* apply to a population and describe social factors that influence performance of a behaviour in that population.

In cultures where breast pumps are not accepted or settings where breast pumps are not easily accessed, use of breastfeeding self-efficacy questionnaire may not be appropriate, as it contains an item about using breast pumps [37]. Theory of planned behaviour has no standard questionnaire [44], thus there were no unified variables to test the theories in the included studies.

*Reasoned action approach* provides an explanation as to why different background factors are related (or are not related) to a particular behaviour [45]. Therefore, it is useful to reduce disparities or increase rates of EBF especially among women who are least likely to achieve their breastfeeding goals.

### Overview of philosophies

*Pragmatism* is an American philosophy first developed by Charles Pierce. It is a way of doing philosophy, it is concerned with actions [46]. *Pragmatism* evaluates the truth of the meaning of theories in terms of the successful application of those theories. That is, theories are meaningful only if they are practically applicable. Pragmatists subscribe to the notion of instrumentalism because they view theories as instruments for problem solving. In pragmatism, the whole of a concept or phenomenon is found in the consequences of the concept or phenomenon [47]. *Phenomenology* is a philosophy developed by Husserl which involves description of lived experience, free from preconceived ideas about the phenomenon. *Phenomenology* attempts to describe experience from the perspective of the person who had the experience first-hand [48]. The difference between *pragmatism* and *phenomenology* is that *pragmatism* attempts to solve a problem using practical methods whereas phenomenology aims to understand the problem/experience [49]. *Pragmatism* has been criticized for its restricted use in identifying and analysing structural social problems [50] whereas *phenomenology* is limited by difficulty its subjectivity and difficulty with data analysis and interpretation [51].

### Overview of frameworks and models

Green’s *proceed-precede model* was first published as an evaluation framework in 1974 [52], as Precede in 1980 [53], and as a full framework in 1991 [54]. *Precede-Proceed* framework comprises eight phases to guide professionals to develop, implement and evaluate health promotion programmes [55], using *socio-ecological model* to assess individual characteristics and socio-political conditions [56]. Bronfenbrenner’s (1977) *socio-ecological model* explained that individuals are influence and are influenced by a complex range of social factors and environmental interactions [57]. The *belief, attitudes, subjective norms and enabling factors (BASNEF) model,* developed by Hubley (1988) originated from Precede model and TRA. It posited that belief, attitude and subjective norms determine behavioural intention, which supports enabling factors for a behaviour [58]. *BASNEF model* has been used to positively influence nutritional behaviours to reduce risk factors for cardiovascular diseases [59]. Similarly, *attitude-social influence-self-efficacy model*, influenced by *TPB, reasoned action approach* and Bandura’s *theory of self-efficacy* and developed by de Vries et al. (1988) suggests that attitude, social influence, and self-efficacy determine behavioural intention which in turn predicts behaviour [60, 61]. *Information-motivation-behavioural-skills (IMB) model* also suggested that health-related information, motivation, and behavioural skills are primary determinants of performance of health behaviours [62]. Nicholson (1990) developed an analytical framework to facilitate adaptation—*transition cycle.* The cycle consisted of four stages including preparation, encounter, adjustment, and stabilization [63]. The stages are useful to enhance readiness, reduce negative emotions, support personal change and role development, and maintain successful adaptation outcomes [64]. In her *model of infant feeding behaviours,* Lutter recognized the importance of self-efficacy in the achievement of a behaviour. The model posited that infant feeding depends on two factors—the interaction between a woman’s choice to breastfeed and her ability to act upon the choice (self-efficacy). Lutter further described that these factors are influenced by three determinants including proximate, intermediate, and underlying determinants. Proximate determinants are primary conditions (maternal choices and ability to act on these choices) that must be present for breastfeeding to occur, these primary conditions are affected by intermediate determinants (information and support) which are in turn influenced by underlying determinants (social norms, socio-demographic characteristics) [65]. Lewin’s *change management model* posits that organizational change occurs in three stages including unfreeze, move/transition, and unfreeze [66].

The primary role of health professionals is to promote health. Thus, the *health promotion model,* developed by Pender (1982) promotes health professionals’ understanding of health behaviour determinants and empowers them to provide quality behavioural counselling [67]. *GATHER* framework (Greet, ask, tell, help, explain and return) is a framework used to provide competent and caring counselling. Moreover, Titler’s *Iowa’s model of evidence-based practice* was developed to empower health professionals to translate research findings into practice to provide quality care [68]. Novak’s *concept mapping*, developed by in 1972 is useful for organization and representation of knowledge. Concept maps illustrate specific label for a concept in a box with lines showing linking words that create a meaningful statement [69]. Further, Bartholomew’s (1998) *intervention mapping* is a framework designed to facilitate the development of health education interventions. The framework has five steps: matrix creation, intervention methods selection, program design, identifying adoption and implementation plans, and program evaluation plan generation [70].

Some models are applicable to systems. *Baby-Friendly Hospital Initiative* launched by World Health Organization (WHO) and United Nations Children’s Fund (UNICEF) to increase support for breastfeeding in hospitals globally included ten steps can be implemented to achieve successful breastfeeding [71]. Similarly, the *social franchise model* for infant and young child feeding (IYCF) suggested that a franchise facility must provide these services—exclusive breastfeeding promotion, support and management, and complementary feeding education and management [72]. Institute of Healthcare Improvement also developed the *breakthrough series (BTS) collaborative model* to bring large number of hospital teams together to seek improvement in a specific topic or field [73]. A common weakness of the system intervention models is their unsuitability to design or evaluate individual-focused interventions.

*Models of infant feeding behaviours, attitude-social influence-self-efficacy, information-motivation-behavioural-skills and BASNEF model* explained individual characteristics that determine performance of a behaviour whereas the other frameworks/models apply to a population. For example, *Baby-Friendly Hospital Initiative, social franchise model, and breakthrough series (BTS) collaborative model* describe actions required from health professionals towards the implementation of interventions to promote health/health behaviour. Though the included studies in this review did not use Lean Six Sigma model, the model is a process improvement model involving five phases: define, measure, improve, analyse, and control. Lean Six Sigma model, which has been successfully used to develop interventions that reduced patient waiting time at clinics [74], may be applied to design system interventions to promote EBF.

## Results

### Characteristics of the studies

The articles selected for this review varied in the study design and the setting in which the studies were conducted (Table 2). Most of the studies were conducted in United States (*n* = 10) and China (*n* = 10), followed by Indonesia (*n* = 4), Iran (*n* = 4), Vietnam (*n* = 3), Australia (*n* = 2), Netherlands (*n* = 2), Egypt (*n* = 1), New Zealand (*n* = 1), Norway (*n* = 1), Turkey (*n* = 1), Malaysia (*n* = 1), Niger (*n* = 1), Thailand (*n* = 1), Mexico (*n* = 1) and Taiwan (*n* = 1). Ten studies were published after 2019, 29 studies were published from 2010 – 2019, and five from 2002—2009. Study designs included randomized control trials (RCT; *n* = 24), correlational (*n* = 7), quasi-experimental (*n* = 5), qualitative (*n* = 5), and mixed methods (*n* = 3).

**Table 2:** Characteristics of included studies

ID	Authors (Year) Country	Design Sample size	Philosophy/framework	Use of philosophy	EBF assessed
01	Ahmadi et al. (2016) [75] Iran	RCT (*n* = 124)	-Hubley’s Belief, Attitudes, Subjective Norms and Enabling factors (BASNEF) model -GATHER steps	Program implementation	Yes
02	Ahmed (2008) [76] Egypt	RCT (*n* = 60)	-Bandura’s social cognitive theory -Green’s PRECEDE model	Program implementation	Yes
03	Alianmoghaddam et al. (2019) [77] New Zealand	Qualitative (*n* = 30)	-Granovetter’s strength of weak ties theory -Milligan and Wiles’ theory of landscapes of care	Description of findings from thematic analysis	No
04	Arbour et al. (2019) [78] USA	Quasi-experimental (n = 16)	-Institute of Healthcare Improvement’s Breakthrough Series (BTS) collaborative model	Program implementation	Yes
05	Baerug et al. (2016) [79] Norway	Quasi-experimental (*n *= 2032)	-Pragmatism -Baby-Friendly Initiative	-Study design -Program implementation	Yes
06	Bai et al. (2007) [80] USA	Qualitative (*n* = 25)	-Ajzen’s theory of planned behaviour	Data collection	No
07	Bai et al. (2011) [81] USA	Cross-sectional survey (*n* = 236)	-Ajzen’s theory of planned behaviour	Data collection	No
08	Bich et al. (2019) [82] Vietnam	Quasi-experimental (*n* = 802)	-Bandura’s social cognitive theory -Ajzen’s theory of planned behaviour	-Program implementation	Yes
09	Blyth et al. (2002) [83] Australia	Prospective survey (*n* = 300)	-Bandura’s self-efficacy theory -Dennis’ breastfeeding self-efficacy theory	-Program content development -Program implementation -Program evaluation: selection of measurements	Yes
10	Brockman (2015) [84] USA	Grounded theory	-Titler’s IOWA model of evidence-based practice -Lewin’s change management model	-Program implementation -Breastfeeding transition monitoring	Yes
11	Bueno-Gutiérrez et al. (2021) [85] Mexico	RCT (*n* = 80)	-Socio-ecological framework	-Program content development -Program implementation	Yes
12	Cangol & Sahin (2017) [86] Turkey	RCT (*n *= 100)	-Pender's Health Promotion Model	-Program content development -Program implementation -Program evaluation: selection of measurements	Yes
13	Chan et al. (2016) [87] China	RCT (*n* = 71)	-Bandura’s self-efficacy theory -Dennis’ breastfeeding self-efficacy theory	-Program content development -Program implementation -Program evaluation: selection of measurements	Yes
14	Froehlich et al. (2020) [88] USA	Mixed methods (*n* = 11)	-Husserl’s Phenomenology -Nicholson’s transition cycle	-Data analysis -Explanation of findings	Yes
15	Ghaffari et al. (2019) [89] Iran	RCT (*n* = 101)	-Ajzen’s theory of planned behaviour	Program implementation	Yes
16	Gijsbers et al. (2006) [90] Netherlands	RCT (*n* = 113)	-De Vries’ Attitude-social influence-self-efficacy-model (ASE model)	Program implementation	Yes
17	Gu et al. (2016) [91] China	Longitudinal RCT (*n* = 285)	-Ajzen’s Theory of planned behaviour	-Program content development -Program implementation	Yes
18	Henry et al. (2017) [92] USA	Mixed methods (*n* = not stated)	-Kotter’s theory of change -Baby-Friendly Initiative	-Initiation of culture change -Addressing knowledge-practice gap	Yes
19	Lestari et al. (2019) [93] Indonesia	Qualitative (*n* = 11)	-Husserl’s Phenomenology	Description of EBF promotion activities	No
20	Liu et al. (2017) [94] China	Quasi-experimental (*n* = 150)	-Bandura’s self-efficacy theory -Dennis’ breastfeeding self-efficacy theory	-Program content development -Program implementation -Program evaluation: selection of measurements	Yes
21	Mccarter-spaudling & Gore (2009) [95] USA	Descriptive longitudinal (*n* = 155)	-Bandura’s social cognitive theory -Dennis’ breastfeeding self-efficacy theory	Measurement breastfeeding self-efficacy	Yes
22	Mcqueen et al. (2011) [96] China	RCT (*n* = 150)	-Bandura’s self-efficacy theory -Dennis’ breastfeeding self-efficacy theory	-Program content development -Program implementation -Program evaluation: selection of measurements	Yes
23	Moussa Abba et al. (2010) [97] Niger	Exploratory qualitative (*n* = 31)	-Lutter’s model of infant feeding behaviour	Basis for observation of dimensions	No
24	Mestsers et al. (2018) [98] Netherlands	RCT (*n* = 113)	-Bartholomew’s Intervention mapping -Bandura’s social cognitive theory	-Program design, implementation, and evaluation -Practical application for inducing change	Yes
25	Nguyen et al. (2014) [99] Vietnam	RCT (*n* = 2045)	-Alive and Thrive Vietnam’s Social Franchise Model	Program implementation	Yes
26	Nguyen et al. (2016) [100] Vietnam	RCT (*n* = 2045)	-Fishbein’s Theory of Reasoned Action Approach	Program implementation	Yes
27	Nichols et al. (2009) [101] Australia	RCT (*n* = 90)	-Bandura’s self-efficacy theory -Dennis’ breastfeeding self-efficacy theory	-Program content development -Program implementation -Program evaluation: selection of measurements	Yes
28	Pollard (2011) [102] USA	RCT (*n* = 86)	-Bandura’s social cognitive theory	-Program implementation	Yes
29	Rahayu (2017) [103] Indonesia	Descriptive correlational (*n* = 30)	-Mercer’s Theory of Maternal Role Attainment	Explanation of findings	No
30	Rasoli et al. (2020) [104] Iran	Quasi-experimental (*n* = 168)	-Extended Ajzen’s theory of planned behaviour	Program implementation	Yes
31	Sadeghi et al. (2021) [105] Iran	RCT (*n* = 52)	-Ajzen’s theory of planned behaviour	-Program content development -Program implementation	Yes
32	Seran et al. (2020) [106] Indonesia	Cross-sectional (*n* = 26)	-Green’s Precede-Proceed Model	Explanation of findings	No
33	Tengku Ismail et al. (2016) [107] Malaysia	Prospective cohort (*n* = 200)	-Ajzen’ theory of planned behaviour	Data collection	Yes
34	Thepha et al. (2019) [108] Thailand	Mixed methods (*n* = 22)	-Novak’s concept mapping	-Study design -Data collection	No
35	Tseng et al. (2020) [109] Taiwan	RCT (*n* = 93)	-Bandura’s self-efficacy theory -Dennis’ breastfeeding self-efficacy theory	-Program content development -Program implementation -Program evaluation: selection of measurements	Yes
36	Tuthill et al. (2017) [110] USA	RCT (*n* = 68)	-Fisher and Fisher’s Information-Motivation-Behavioural-Skills model -Dennis’ breastfeeding self-efficacy theory	-Program implementation -Program evaluation: selection of measurements	Yes
37	Wambach et al. (2011) [111] USA	RCT (*n* = 287)	-Ajzen’s Theory of planned behaviour -Mann’s adolescent decision-making (ADM) competence theory	Treatment group received breastfeeding education intervention based on TPB and ADM	Yes
38	Wan et al. (2016) [112] China	Longitudinal RCT (*n* = 285)	-Ajzen’s Theory of planned behaviour	Program implementation	Yes
39	Wen et al. (2021) [113] China	RCT (*n* = 132)	-Ajzen’s Theory of planned behaviour	Program implementation	Yes
40	Wu et al. (2014) [114] China	RCT (*n* = 74)	-Bandura’s self-efficacy theory -Dennis’ Breastfeeding self-efficacy theory	Treatment group received breastfeeding self-efficacy intervention	Yes
41	You et al. (2020) [115] China	RCT (*n* = 226)	-Bandura’s self-efficacy theory -Dennis’ breastfeeding self-efficacy theory	-Program content development -Program implementation -Program evaluation: selection of measurements	Yes
42	Yunitasari et al. (2020) [116] Indonesia	Descriptive correlational (*n* = 221)	-Pender’s Health promotion model	Explanation of findings	No
43	Zhang et al. (2021) [117] China	RCT (*n* = 140)	-Ajzen’s theory of planned behaviour	-Program content development -Program implementation	Yes
44	Zhu et al. (2017) [118] China	RCT (*n* = 285)	-Ajzen’s theory of planned behaviour	-Program content development -Program implementation	Yes

Almost 9500 mother–child pairs and family pairs participated in the 44 studies. The sociodemographic characteristics were reported in 42 studies. Participants ranged from only mothers (*n* = 35), mother-infant pairs (*n* = 3), family (*n* = 2), health professionals (*n* = 2) and hospitals (*n *= 2). No study included fathers only or extended family. The setting of the articles ranged widely from the hospital [2], prenatal/maternity clinics (*n* = 33) primary health clinics [6], Local Implementing Agencies (LIAs) (ID-05) (*n* = 1) and communities [2]. Thirty-five studies assessed the prevalence of postpartum EBF at different time intervals while nine studies suggested measures to promote EBF. Most studies reported EBF at the individual level, only three studies reported at the family and hospital levels. Forty-two studies included term/healthy infants while two studies included preterm infants [75, 76].

### Application of theories/philosophies/frameworks to exclusive breastfeeding promotion

Ajzen’s *theory of planned behaviour* (*n* = 13) and Dennis’ *breastfeeding self-efficacy theory* (*n* = 10) were the most used theories in the studies [77–84]. Findings from this review suggests that EBF programmes oriented within theories are effective in increasing EBF rates. While EBF rates increased in all included studies, statistically significant increase at 6 months were reported in few studies. For example, intervention groups had higher EBF rates compared with control groups in studies that applied *theories of breastfeeding self-efficacy*—37% vs. 14% [78], 32% vs. 14% [82], 56% vs. 37% [83], *planned behaviour*—2% vs. 0% [85], 42% vs. 10% [86, 87], 88% vs. 77% [88], *reasoned action approach*- (72% vs 63%) [89], *intervention mapping-* (48% vs 27%) [90], *social franchise model-* (62% vs 40%) [91], *attitude-social influence-self-efficacy model-* (48% vs 27%) [92], and *Baby-Friendly Hospital Initiative* (18% vs. 14%) [93]. *Theories of breastfeeding self-efficacy and planned behaviour* have been tested to support and protect exclusive breastfeeding. Chipojola et al. (2020) tested the overall effects of both theories on EBF and reported significant increase in EBF rates in intervention group compared with control group across studies included in their review and meta-analysis [94].

*Theories of self-efficacy and planned behaviour* are useful for data collection, program content development and implementation. Dennis’ breastfeeding self-efficacy questionnaire in its short form [95] measured breastfeeding self-efficacy in women during pre-partum and/or postpartum and assessed the effect of an intervention on breastfeeding self-efficacy [96]. *Social cognitive theory* was used to select suitable educational strategies to promote EBF among women with preterm infants [76]. Moussa Abba et al. (2010) used the *model of infant feeding behaviours* to identify breastfeeding [97]. TPB was used to guide the design of study interventions, design questionnaires, predict and explain breastfeeding outcomes [87, 98, 99]. *Reasoned action approach* was used to design study interventions- interpersonal counselling and exposure to mass media- to promote EBF practices in Vietnam [89]. The *Attitude-social influence-self-efficacy model* was used to develop an educational programme (intervention) [92]. Likewise, Pender’s *health promotion model* was used in included studies to design an intervention—breastfeeding motivation program [100] and explain research findings [101]. *Information-motivation-behavioural-skills model* was used to design counselling sessions that focused on enhancing IMB breastfeeding determinants among HIV-infected women [102]. *Mann’s Adolescent decision-making competence theory* was used to design developmentally sensitive, education and counselling intervention [103].

Unlike theories of self-efficacy and planned behaviour that are primary based on maternal variables, social theories and theories for system interventions explain the influence of societal interactions/structures on exclusive breastfeeding. Alianmoghaddam and colleagues used the theories of *strength of weak ties and landscapes of care* to explain importance of social relationships, social interactions and social support within virtual communities that are associated with breastfeeding [104]. Support systems for women were identified using the *theory of maternal role attainment* [105]. *Social Franchise Model* was used to design breastfeeding intervention—infant and young child feeding (IYCF) counselling services [91]. Similarly, *Breakthrough Series (BTS) collaborative model* guided the planning of a programme—Home Visiting Collaborative Improvement and Innovation Network (HV CoIIN)—which increased EBF duration [106]. Titler’s *Iowa’s model of evidence-based practice* guided the implementation of a new in-patient model of nursing care—mother–child dyad care [107]. Lewin’s *change management model* to manage the complex change processes in the transition from the traditional model of obstetric nursing to care of mother–child dyad. *Kotter’s change theory* was used to initiate culture change for a successful adoption of *Baby-Friendly Hospital Initiative* [108] which was adopted in community health service in another study and it significantly increased EBF rates in intervention [93].

Frameworks were also used for program development. Ahmed (2014) used the *Precede model* to design a five-session breastfeeding educational program [76] and explain family support factors that promoted exclusive breastfeeding rates [109]. Ahmadi et al. (2016) used *BASNEF model* to design questionnaire about breastfeeding attitude of women; the questionnaire had reliability score (Cronbach’s alpha) of 0.7. *GATHER* (Greet, ask, tell, help, explain and return) steps was also used to guide breastfeeding consultation sessions for the intervention group [75]. Transition cycle was used to illustrate and explain mothers’ transition to breastfeeding after childbirth [110]. *Concept mapping* was used during all three intervention meetings to provide information and findings regarding identifying and prioritising facilitators and barriers to 6-month exclusive breastfeeding [111]. Similarly, *intervention mapping* was used as a concept map to guide development of educational program [90].

Lastly, philosophies guided study designs and data collection. Baerug et al. (2016) used pragmatism as the basis for their quasi-randomized control trial study which examined the effect (consequence) of baby-friendly community health services on EBF [93]. On the other hand, phenomenology was used to describe participants’ involvement in EBF promotion activities [112], qualitatively analyse data collected from participants and to formulate essence descriptors of their breastfeeding experiences and daily routine [110].

## Discussion

The objective of this scoping review was to identify philosophical schools of thoughts and theories that guide research on promoting exclusive breastfeeding practice. The scoping review clearly established that a wide range of different interventions based on philosophies and theories are effective to promote exclusive breastfeeding practice for both healthy full-term and preterm infants. Theories of self-efficacy and planned behaviour were the most common theories that significantly increased EBF rates at 6 months [78, 82, 83, 85–88]. Chipojola et al. (2020) reported similar finding and recommended the use of these two theories to design interventions in future studies to increase exclusive breastfeeding rates [94]. Philosophies provided the basis to explore different methods that may promote the practice of exclusive breastfeeding [93, 110, 112]. Whilst self-efficacy theories were used for intervention implementation and evaluation at individual levels [80, 81], theories for systems intervention provided a larger context to examine effect of interventions on breastfeeding exclusivity [93, 106]. Further, social theories provided opportunity to modify variables in the environment and test the influence of the modification on exclusive breastfeeding rates [91, 105]. Thus, researchers may choose theories from these categories depending on the scope of their studies. The theory of planned behaviour was used primarily to implement interventions [86, 87, 103]. Whereas frameworks provided step-by-step instructions for program development and implementation [75, 76, 101] to ensure accurate implementation of interventions and provision of a foundation for evaluation of the interventions. The use of a framework/model to guide a study is limited as the included frameworks have several stages, but most studies need to implement only a few stages to meet their goals. Thus, limiting the generalizability of the frameworks across studies.

Some theories and frameworks were effective at promoting EBF among women who may be unable to achieve their breastfeeding goals. For example, TPB significantly increased EBF among women with low rates (30%) of EBF [86]. Similarly, Dennis’ theory of breastfeeding self-efficacy significantly increased EBF rates among African American women [96]. Bandura’s social cognitive theory was used to design an educational intervention which significantly increased EBF rates among women with preterm infants [76]. Kotter’s theory of change facilitated successful implementation of the baby-friendly hospital initiative which increased EBF among Latina women reported to be the most likely population to supplement early with formula due to perceived milk insufficiency [108]. Likewise, implementation of the baby-friendly hospital initiative increased EBF rates among women in rural and semi-urban districts in Norway [93].

Overall, TPB was the most used theory that significantly increased exclusive breastfeeding rates at 6 months [85–88]. A reason for the frequent use of TPB may be its effectiveness at predicting behaviours and its usefulness in the development of educational programs or interventions. Indeed, Bai et al. (2019) reported in their critical review of theories supporting breastfeeding that based on the holistic effects of its propositions, TPB is more applicable to promote breastfeeding compared with Dennis’ breastfeeding self-efficacy theory, and Bandura’s self-efficacy and social cognitive theories [113]. Further, breastfeeding self-efficacy theory is limited by the interaction between self-efficacy and previous breastfeeding experience, which may have biased the actual effectiveness of the theory on EBF. McCarter-Spaulding and Gore (2009) reported that breastfeeding self-efficacy scores were higher among mothers who had previous breastfeeding experience [96]. TPB posited that perceived behavioural control and behavioural intention can be used to directly predict behavioural achievement [114]. Behavioural intention has three conceptually different determinants including *attitude towards the behaviour*- the extent to which a person has favourable or unfavourable evaluation of a specific behaviour-, *subjective norm*- perceived social pressure to perform a behaviour or not-, and *perceived behavioural control* – perceived ease or difficulty of performing a behaviour [115]. Perceived behavioural control on the other hand is assumed to reflect past experiences and anticipated challenges regarding performing a behaviour [114]. TPB is used to predict a behaviour based on two conditions- perceived behavioural control and behavioural intention. These two conditions may also be referred to as antecedents. The application of TPB in research to determine the effect of interventions implies testing the accuracy of the theory’s scientific prediction. Scientific prediction attempts to determine the effect of the initial conditions, otherwise referred to as antecedents/independent variables on specific dependent variables [116]. Hempel posited that a prediction is valid if it has logical and empirical adequacy [117]. That is, the explanans (premises) must contain at least one law of nature and the statements constituting the explanans must be true (empirically verified). Empiricists believe in verifiability, the only valid source of knowledge for them is empirical experience- what is perceived through the senses [118]. Therefore, they posited that a statement is meaningful only if it has been proven true or false through means of experience (experiment). Empirical verification can be achieved through scientific method, experimentation, or laboratory science. TPB was tested in previous studies and found to successfully predict dishonest actions [119], leisure behaviours [120], and implement interventions that will be effective to change behaviours [121]. Thus, propositions in TPB have been empirically verified, which may be another reason for its frequent use in the included studies.

### Strength and limitations

Scoping reviews allow for more quality result than systematic review, because unlike the latter, it allows for identification of relevant studies irrespective of study designs [23]. To our knowledge, this is the first scoping review to map evidence specific to philosophies and/or frameworks used to address exclusive breastfeeding promotion. The review used rigorous and transparent methods throughout the study. Theories identified in this review are similar with those identified in previous studies [113, 122]. Notwithstanding, this review included additional frameworks and theories that used decision-making and developmental models. Compared with other scoping reviews, this study included relatively large number of articles accessed from different databases. Hence, results of this scoping review have enabled development of specific search strategies for future reviews. However, our review may not have identified all studies in the literature, particularly studies that applied philosophical schools of thought to exclusive breastfeeding promotion, as most included articles were theory-based. Additionally, the culture in settings of included studies should be considered when selecting a theory/philosophy for future studies, as it may influence the effectiveness of the theory/philosophy. Hence, future studies may test theories and/or instruments developed from these theories to achieve effective cross-cultural adaptation.

## Conclusions

This study established that strategies supported by philosophies and theories are useful to increase exclusive breastfeeding rates, especially in interventions involving breastfeeding education, empowerment, and counselling. Theories of planned behaviour and self-efficacy are useful to design and implement these interventions. We recommend that future studies aimed at reducing disparities in exclusive breastfeeding rates adopt theories of breastfeeding self-efficacy, planned behaviour, and social cognitive theory as these theories significantly increased exclusive breastfeeding among women that are least likely to breastfeed. Future scoping reviews should include comprehensive search of more databases to access and include more studies that use philosophical schools of thought to promote exclusive breastfeeding practice.

## Supplementary Information

**Additional file 1.** 

## Data Availability

All data generated or analysed during this study are included in this published article [and its supplementary information files].

## Abbreviations

ADM: Adolescent decision-making
BASNEF: Belief, Attitudes, Subjective Norms and Enabling factors
EBF: Exclusive breastfeeding
GATHER: Greet, Ask, Tell, Help, Explain and Return
TPB: Theory of Planned Behaviour
WHO: World Health Organization
