# Supplementary material for: Promoting the practice of exclusive breastfeeding: a philosophic scoping review
Source: BMC Pregnancy Childbirth. 2022 May 1;22:380. doi: 10.1186/s12884-022-04689-w (PMC9063230; doi:10.1186/s12884-022-04689-w)
Supplement: Supplementary file 1 — Additional file 1. [file 12884_2022_4689_MOESM1_ESM.docx]

**Table 3** CASP Randomized Controlled Trial Standard Checklist

| Study | Did the study address a clearly focused research question? | Was the assignment of participants to interventions randomised? | Were all participants who entered the study accounted for at its conclusion? | Were participants/investigation/people assessing outcome(s) blinded? | Were the study groups similar at the start of the randomised controlled trial? | Apart from the experimental intervention, did each study group receive the same level of care? | Were the effects of intervention reported comprehensively? | Was the precision of the estimate of the intervention or treatment effect reported? | Do the benefits of the experimental intervention outweigh the harms and costs? | Can the results be applied to your local population? | Would the experimental intervention provide value to the people in your care? | Score  (out of 11) |
| --- | --- | --- | --- | --- | --- | --- | --- | --- | --- | --- | --- | --- |
| Ahmadi et al (2016) (75) | X | X | X | X | X | - | X | X | X | X | X | 10 |
| Ahmed (2008)  (76) | X | X | X | - | X | - | X | X | X | X | X | 9 |
| Arbour et al (2019)  (78) | X | - | X | - | X | - | X | X | X | X | X | 8 |
| Baerug et al (2016)  (79) | X | - | X | - | X | - | X | X | X | X | X | 8 |
| Bich et al (2019) (82) | X | X | X | - | X | - | X | X | X | X | X | 9 |
| Bueno-Gutiérrez et al (2021) (85) | X | X | X | - | X | X | X | X | X | X | X | 10 |
| Cangol & Sahin (2017) (86) | X | X | X | - | X | X | X | X | X | X | X | 10 |
| Chan et al (2016) (87) | X | X | X | - | - | X | X | X | X | X | X | 9 |
| Ghaffari et al (2019) (89) | X | X | X | - | X | - | X | X | X | X | X | 9 |
| Gijsbers et al (2006) (90) | X | X | X | - | X | - | X | X | X | X | X | 9 |
| Gu et al (2016) (91) | X | X | X | X | X | X | X | - | X | X | X | 10 |
| Liu et al (2017) (94) | X | - | X | - | X | X | X | - | X | X | X | 8 |
| Mcqueen et al (2011) (96) | X | X | X | X | X | - | - | X | X | X | X | 9 |
| Mestsers et al (2018) (98) | X | X | X | X | X | X | X | - | X | X | X | 10 |
| Nguyen et al (2014) (99) | X | X | X | X | X | X | X | - | X | X | X | 10 |
| Nguyen et al (2016) (100) | X | X | X | X | X | - | X | X | X | X | X | 10 |
| Nichols et al (2009) (101) | X | X | X | X | X | X | X | - | X | X | X | 10 |
| Pollard (2011) (102) | X | X | X | - | X | X | X | - | X | X | X | 9 |
| Rasoli et al (2020) (104) | X | - | X | - | - | X | X | X | X | X | X | 8 |
| Sadeghi et al (2021) (105) | X | X | X | - | X | X | X | - | X | X | X | 9 |
| Tseng et al (2020) (109) | X | X | X | X | X | X | X | - | X | X | X | 10 |
| Tuthill et al (2017) (110) | X | X | X | X | X | X | X | - | X | X | X | 10 |
| Wambach et al (2011) (111) | X | X | X | X | X | X | X | X | X | X | X | 11 |
| Wan et al (2016) (112) | X | X | X | X | X | X | X | - | X | X | X | 10 |
| Wen et al (2021) (113) | X | X | X | - | X | X | X | X | X | X | X | 10 |
| Wu et al (2014) (114) | X | X | X | X | X | X | X | - | X | X | X | 10 |
| You et al (2020) (115) | X | X | X | X | X | X | X | - | X | X | X | 10 |
| Zhang et al (2021) (117) | X | X | X | - | - | X | X | - | X | X | X | 8 |
| Zhu et al (2017) (118) | X | X | X | - | X | X | X | - | X | X | X | 9 |

**Table 4** Appraisal table for other quantitative studies

| Study | What is the statement of the aim of the data collection? | Does method seem suitable given the aim of the study? | What was the method used to collect the data? | Is the method of processing and analysing the results described in the methods section? | On how many people are the results based? | Did an ethics committee approve the study? | What were the large results that relate to the aim of the study? | Did they give a clear answer to their aim? | What were the strengths and limitations? | How do the results relate to practice? | Score (out of 10) |
| --- | --- | --- | --- | --- | --- | --- | --- | --- | --- | --- | --- |
| Bai et al (2011) (81) | X | X | X | X | X | X | X | X | X | X | 10 |
| Blyth et al (2002) (83) | X | X | X | X | X | X | X | X | X | X | 10 |
| Mccarter-spaudling & Gore (2009) (95) | X | X | X | X | X | X | X | - | X | X | 9 |
| Rahayu (2017) (103) | X | X | X | X | X | - | X | - | X | X | 8 |
| Seran et al (2020) (106) | X | X | X | X | X | X | X | X | - | X | 9 |
| Tengku Ismail et al (2016) (107) | X | X | X | X | X | X | X | X | X | X | 10 |
| Yunitasari et al (2020) (116) | X | X | X | X | X | X | X | - | - | X | 8 |

**Table 5** CASP checklist assessment table for Qualitative studies

| Study | Was there a clear statement of the aims of the research? | Is a qualitative methodology appropriate? | Was the research design appropriate to address the aims of the research? | Was the recruitment appropriate to the aims of the research? | Was the data collected in a way that addressed the research issue? | Has the relationship between researcher and participants been adequately considered? | Have ethical issues been taken into consideration? | Was the data analysis sufficiently rigorous? | Is there a clear statement of findings? | Is the research valuable? | Score (out of 10) |
| --- | --- | --- | --- | --- | --- | --- | --- | --- | --- | --- | --- |
| Alianmoghaddam et al (2019) (77) | X | X | X | X | X | - | X | X | X | X | 9 |
| Bai et al (2007) (80) | X | X | X | X | X | - | X | X | X | X | 9 |
| Brockman (2015) (84) | X | X | X | X | - | - | X | X | X | X | 8 |
| Froehlich et al (2020) (88) | X | X | X | X | X | - | X | X | X | X | 9 |
| Henry et al (2017) (92) | X | X | X | X | X | X | X | X | X | X | 10 |
| Lestari et al (2019) (93) | X | X | X | X | X | X | X | X | X | X | 10 |
| Moussa Abba et al (2010) (97) | X | X | X | X | X | X | X | X | X | X | 10 |
| Thepha et al (2019) (108) | X | X | X | X | X | - | X | X | X | X | 9 |
